# ERp44 is required for endocardial cushion development by regulating VEGFA secretion in myocardium

**DOI:** 10.1111/cpr.13179

**Published:** 2022-01-28

**Authors:** Youkun Bi, Zhiguang Yang, Meng Jin, Kui Zhai, Jun Wang, Yang Mao, Yang Liu, Mingqin Ding, Huiwen Wang, Fengchao Wang, Hong Cai, Guangju Ji

**Affiliations:** ^1^ Key Laboratory of Interdisciplinary Research Institute of Biophysics Chinese Academy of Sciences Beijing China; ^2^ University of Chinese Academy of Sciences Beijing China; ^3^ National Institute of Biological Sciences Beijing China; ^4^ Department of Dermatology Air Force Medical Center PLA Beijing China

## Abstract

**Objectives:**

Endocardial cushions are precursors of the valve septum complex that separates the four heart chambers. Several genes have been implicated in the development of endocardial cushions. Specifically, ERp44 has been found to play a role in the early secretory pathway, but its function in heart development has not been well studied.

**Materials and Methods:**

In this study, we established conditional and tissue‐specific knockout mouse models. The morphology, survival rate, the development of heart and endocardial cushion were under evaluation. The relationship between ERp44 and VEGFA was investigated by transcriptome, qPCR, WB, immunofluorescence and immunohistochemistry.

**Results:**

ERp44 knockout (KO) mice were smaller in size, and most mice died during early postnatal life. KO hearts exhibited the typical phenotypes of congenital heart diseases, such as abnormal heart shapes and severe septal and valvular defects. Similar phenotypes were found in *cTNT*‐*Cre* ^+/−^; *ERp44^fl^* ^/^ *^fl^* mice, which indicated that myocardial ERp44 principally controls endocardial cushion formation. Further studies demonstrated that the deletion of ERp44 significantly decreased the proliferation of cushion cells and impaired the endocardial‐mesenchymal transition (EndMT), which was followed by endocardial cushion dysplasia. Finally, we found that ERp44 was directly bound to VEGFA and controlled its release, further regulating EndMT.

**Conclusion:**

We demonstrated that ERp44 plays a specific role in heart development. ERp44 contributes to the development of the endocardial cushion by affecting VEGFA‐mediated EndMT.

## Graphical abstract

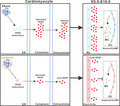

## 1 INTRODUCTION

Congenital heart defect (CHD) has been defined as anatomic dysplasia of the heart or great vessels during intrauterine development, irrespective of the age at presentation.^1^ The endocardial cushion (EC) is a transitional structure during heart development, which only appears in the atrioventricular tube and outflow tract of the embryonic heart, and later develops into the compartment and valves of the atrioventricular and outflow tract, respectively.^2^ Abnormal endocardial cushion development is directly responsible for CHD. Morphologically, the normal extracellular matrix secreted by the myocardium promises the normal development of EC.^3^, ^4^ Many genes have been shown to play essential roles in endocardial cushion development, including molecules involved in transcription, epigenetics, adhesion and migration, such as TGF‐β2, Wnt3, VEGF.^5^ These cytokines induce the depolarization of the endothelial cells in the endocardial cushion area and weaken the connections between cells, resulting in the migration of mesenchymal cells (MSCs) to the myocardium. The proliferated MSCs together contribute to forming EC. The above process is named the endothelial‐mesenchymal transition (EMT).^6^ Although the effect of EMT, to some extent, has been clarified on the EC development, additional details remain to be elucidated given the complexity and importance to regenerative or therapeutic purposes.

ERp44 is a pH‐regulated chaperone and belongs to the protein disulphide isomerase family.^7^ It contains three thioredoxin domains, a, b and b’, and a flexible carboxy‐terminal tail.^8^ The CRFS motif in the domain is thought to form a disulphide bridge with ERp44 client proteins.^9^, ^10^, ^11^ This flexible tail masks the substrate‐binding site; thus, the protein is in the off‐state at the neutral pH of the endoplasmic reticulum (ER) and in the on‐state at the lower pH of the cis‐Golgi apparatus, allowing it to capture KDEL receptors.^12^, ^13^ In this manner, ERp44 orchestrates the balance between client protein retention in the ER and their secretion by covalent interactions.^14^, ^15^ Several important secretory factors are reportedly regulated by ERp44, including IgM,^16^, ^17^ adiponectin,^18^ SUMF1^19^ and serotonin.^20^, ^21^ Moreover, ERp44 has been implicated in calcium homeostasis. Specifically, ERp44 binds directly to the L3V of inositol 1,4,5‐trisphosphate receptor type 1 (IP_3_R1) to inhibit its activity.^22^ C160/C212, but not C29, participates in the regulation of IP_3_R1.^23^ Huang et al reported that ERp44 may also regulate IP_3_R2.^24^ Recently, Wang et al reported that ERp44 mutant leads to embryonic lethality using mouse model, but they did not provide a direct reason for this phenomenon.^25^ Moreover, Hisatsune et al reported that ERp44 regulates blood pressure.^26^

In the present study, we report that the deletion of ERp44 leads to congenital heart defects. Specifically, a loss of ERp44 causes AVC dysplasia arising from the aberrant proliferation of cushion cells and reduced EndMT by directly regulating VEGFA during heart development. Thus, our findings revealed a critical role for ERp44 in endocardial cushion development.

## 2 METHODS

### 2.1 Mouse breeding and genotyping

All animal studies were performed according to the relevant guidelines and regulations approved by the Committee on Animal Care of the Institute of Biophysics at the Chinese Academy of Sciences in China. Mice were mated at 5:00 pm, and E0.5 was defined as 9:00 am of the next day when the vaginal plug was detected. Genotyping procedure was performed as previously described.^27^ According to the 3R’ principles, all animal‐relevant experiments should be designed to reduce the number of animals used to meet scientific objectives.^28^

### 2.2 Generation of ERp44 conventional knockout mice

ERp44 knockout mice were generated by gene targeting the ESCs of the 129 mouse strain and subsequently injecting positive cells into C57BL/6 blastocysts. A Loxp‐neomycin‐Loxp cassette with a homologous arm was used to replace exon 2 and exon 3 of the *ERp44* gene. The Loxp‐neomycin‐Loxp cassette was deleted in mutant mice by crossing them with CMV‐Cre mice. 129 × C57BL/6 genetic background mice were backcrossed to C57BL/6 for at least 5 generations. For genotyping, a set of two primers was designed within or without exon 2 and exon 3 for both ERp44‐WT‐F/R and ERp44‐G‐F/R. The sequences of the genotyping primers are listed in Table S1.

### 2.3 Generation of ERp44 conditional knockout mice

The *gRNA* (GTTTTAGAGCTAGAAATAGC) sequence was designed to help Cas9 cut DNA. The sgRNA with T7 promoter was transcribed in vitro using the MEGAshortscript™ Kit (Thermo Fisher, Cat No. AM1354). *Cas9* (Addgene, Cat No. 41815) including the T7 promoter was transcribed using the mMACHINE T7 ULTRA Kit (Thermo Fisher, Cat No. AM1345) and purified using the MEGAclear Kit (Thermo Fisher, Cat No. AM1908). The targeting donor was designed to replace exon 2 in the wild‐type allele with two flanking loxP sequences and two homologous arms. gRNA, Cas9 and donor DNA were microinjected into C57BL/6 zygotes and transplanted into pseudopregnant mice. The founder was genotyped with primers against sequence‐F/R, which detected the left loxP sequence, and screen‐F/R, which spanned two loxP sequences. The genotype was then verified with Sanger sequencing. The sequences of relative primers are listed in Table S1.

### 2.4 Quantitative real‐time PCR (qPCR)

Total RNA was isolated with TRIzol, and cDNA was synthesized with the PrimeScriptTM RT Reagent Kit and gDNA Eraser (TaKaRa, Cat No. RR037A). Real‐time PCR was performed using the SYBR Green PCR Mastermix (Solarbio, Cat No. SR1120) and a Rotor‐Gene‐Q instrument (Qiagen). The fold change in target expression between WT and KO mice was calculated using the 2^−ΔΔCt^ method,^29^ and GAPDH was used as an internal reference. The primers are shown in Table S2.

### 2.5 Immunohistochemistry and immunofluorescence

The embryos were excised in cold PBS (10 g/L NaCl, 0.25 g/L KCl, 1.44 g/L Na_2_HPO_4_, 0.25 g/L KH_2_PO_4_) and fixed in 4% paraformaldehyde/PBS at 4°C. Gradually dehydrated embryos were carefully embedded in wax under a stereoscope. H&E staining and Alcian blue staining (Abcam, Cat No. ab150662) were performed according to a standard procedure. Apoptosis was detected with the In Situ Cell Death Detection Kit (Roche, Cat No. 12352200) according to the product manual. Images were acquired using a Leica SCN400 Slide Scanner.

For immunofluorescence, frozen sections (8 μm) or adherent cells were fixed (4% paraformaldehyde/PBS) for 10 min, and antigens on paraffin sections (5 μm) were unmasked with 10 mM sodium citrate buffer. The sections were then washed twice with PBS and permeabilized with 0.3% Triton‐100/PBS for 10 min before being incubated with blocking solution (Beyotime, P0260) for 1 h at room temperature. The sections were then incubated with primary antibodies overnight at 4℃. Subsequently, the sections were washed three times and then incubated with a suitable secondary antibody for 1 h at room temperature. Nuclei were counterstained with 1 µg/ml DAPI (Beyotime, Cat No. C1005), and images were acquired using a Leica SP5 confocal microscopy. Antibodies used in the study and their dilution concentration are listed in Supplementary materials, and all of them were diluted in PBS with 0.3% Triton X‐100 and 1% BSA.

### 2.6 AV cushion explant assay

The AV cushion explant assay was modified from the previous reports.^30^ Briefly, endocardial cushions from the AV cushion were explanted on rat tail collagen gel (Thermo Fisher, Cat No. A1048301). After overnight incubation, the medium was added (M199, Gibco; 1% FBS, Invitrogen; 0.1% ITS, Gibco; 100 U/ml penicillin, 100 mg/ml streptomycin, INALCO), and elongated or spindle‐shaped mesenchymal cells were counted after 48 h and analysed according to Xiong et al.^31^ The degree of EndMT of a given explant was assessed based on the number of mesenchymal cells relative to the mean in all control groups, which was defined as 100% EndMT.

### 2.7 DNA constructs

Full‐length hVEGFA165 cDNA was amplified from A549 cells and ligated into *pcDNA4* to generate *pcDNA4*‐*hVEGFA165* (VEGFA165), *pcDNA4*‐*hVEGFA165*‐*myc* (VEGFA165‐myc) and *pcDNA4*‐*N101Y*‐*myc* (N101Y‐myc, N101Y mutant of VEGFA165). The ERp44 expression vectors, *pcDNA3*.*1* (as control), *pcDNA3*.*1*‐*HA*‐*ERp44* (HA‐WT), *pcDNA3*.*1*‐*HA*‐*C29S* (HA‐C29S, C29S mutant of ERp44) and *pcDNA3*.*1*‐*HA*‐*△T* (HA‐△T, C‐terminal truncation of ERp44), were described previously.^23^

### 2.8 Establishment of cell lines

VEGFA165‐myc amplified from *pcDNA4*‐*hVEGFA165*‐*myc* was ligated into a PQCXIP vector to generate *pQCXIP*‐*VEGFA165*‐*myc*. Then together with VSV‐G and pHIT, they were transfected into Plat E cells for retrovirus packaging. Quality‐checked retroviruses infected HEK‐293T cells supported by polybrene for follow‐up puromycin screening until obtaining modified HEK‐293T cell line stably expressing VEGFA165‐myc. Details of the above procedure referred to previous documents,^32^, ^33^ and it also guided the establishment of the H9C2 cell line overexpressing ERp44 (ERp44 OE).

As far as generating ERp44 knockout (KO) H9C2, ERp44‐sgRNA‐F and ERp44‐sgRNA‐R (Table S3) were annealed, and products were welded into *Lenti*‐*CRISPR*‐*v2* vector digested by *BsmB* I to generate *Lenti*‐*CRISPR*‐*v2*‐*ERp44*‐*shRNA*. This plasmid together with pMD‐2G and pAX2 was transfected into HEK‐293T cells for lentivirus packaging. Followed by virus infection and puromycin screening, an H9C2 cell line with ERp44 KO was established.

### 2.9 Western blot

For the Western blot analysis, the prepared samples were homogenized in and separated by sodium dodecyl sulphate‐polyacrylamide gel electrophoresis (SDS‐PAGE), transferred onto nitrocellulose membranes (Thermo Fisher, Cat No. LC2002) and incubated with primary antibodies (Abcam, UK) against the specific protein. Thereafter, the membranes were incubated with the appropriate horseradish peroxidase (HRP)–conjugated secondary antibodies (1: 10,000, ZSGB‐BIO, China), and the signals were detected using the SuperLumina ECL HRP Substrate Kit (Abbkine, Cat No. K22030). β‐Actin or GAPDH was used as an internal reference to assess protein levels. Three independent repetitive trials were performed.

### 2.10 Transfection and co‐immunoprecipitation (Co‐IP)

HEK‐293T cells were cultured in high‐glucose Dulbecco's modified Eagle's medium containing 10% foetal bovine serum, 100 U/ml penicillin and 0.1 mg/ml streptomycin (culture medium). Around 10^5^ cells were seeded in each well of a 6‐well plate for next‐day transfection with TurboFect™ (Thermo Fisher, Cat No. R0533). The cells were washed twice with PBS, and 2 ml fresh culture medium was added after 12‐h transfection for another 48‐h culture. Then, the culture medium and cells were homogenized and lysed in cold RIPA buffer in the presence of 1 mM PMSF and protease inhibitor cocktail (Cell Signaling Technology, Cat No. 5871S) for 30min. These samples were subjected to centrifugation for gathering supernatant. The prepared samples were mixed with 5 × SDS loading buffer (Beyotime, Cat No. P0015L) and boiled for 10 min for the next Western blot analysis.

For the detection of endogenous Co‐IP analysis, suitable heart tissue was homogenized and lysed in cold NP‐40 buffer (PBS containing 100 mM NaCl, 10 mM NEM, 0.5% NP‐40, 10 mM Tris‐HCl 7.4, protease inhibitor cocktail and PMSF) for 30 min, then centrifuged at 1,2000 r/min for 10 min to collect supernatant, and part of them was used as input. The prepared samples were incubated with ERp44 antibody at 4℃ for 5 h. Then, the mixture was added with ethanol‐free protein‐A/G PLUS‐Agarose beads (Santa, Cat No. SC‐2003) for another 5‐h incubation at 4℃. The reactant above was washed with NP‐40 for 3 times and discarded the supernatant by centrifugation at 1000 *g* for 5 min. SDS loading buffer was used to resuspend washed beads, followed by boil and centrifugation to separate supernatant for Western blot analysis.

For the exogenous Co‐IP experiment, 293T cells grown in 6‐well plates were co‐transfected with VEGFA165 and ERp44 expression vectors or the VEGFA‐myc overexpression vector. After 48–72 h, the cells were washed with PBS and then exposed to 1.2 mM DSP (Pierce) in PBS for 30 min at RT. After quenching with 25 mM Tris‐HCl in PBS, the cells were collected in NP‐40 buffer followed by ultrasonic lysis and centrifugation. The lysates were incubated with anti‐HA or anti‐myc mouse monoclonal antibody agarose resin (CWBIO, Cat No. CW0010) overnight at 4°C. The beads were washed three times with NP‐40 buffer, and the protein was eluted with 0.2 M glycine (pH 2.5), neutralized with 1.5 M Tris‐HCl (pH 9.0). Subsequently, 5 × SDS loading buffer was added and boiled for 10 min to denature the protein.

### 2.11 RNA‐seq and bioinformatic analysis

The total RNA was extracted from the heart AV cushion tissues of E9.5 according to the protocol of RNA‐ease Isolation Reagent (Vazyme, Cat No. R701‐01). After quality quantification, the total RNA was converted to cDNA library and performed with RNA sequencing by BGI Genomics Co., Ltd. The raw data were subjected to ggplot2 assay in R software (version 3.6.1) for describing advanced volcano plot. Identified genes were mapped to Gene Ontology (GO) terms to determine their biological and functional properties.

### 2.12 Statistical analysis

Experiments were performed at least three independent biological replicates for each group. Differences were considered significant at ^*^ *p* < 0.05, ^**^ *p* < 0.01 and ^***^ *p* < 0.001 using one‐way ANOVA or Student's *t* test. Unless otherwise indicated, all results are expressed as mean ± SEM.

## 3 RESULTS

### 3.1 Generation and characterization of ERp44 knockout mice

**FIGURE 1:**
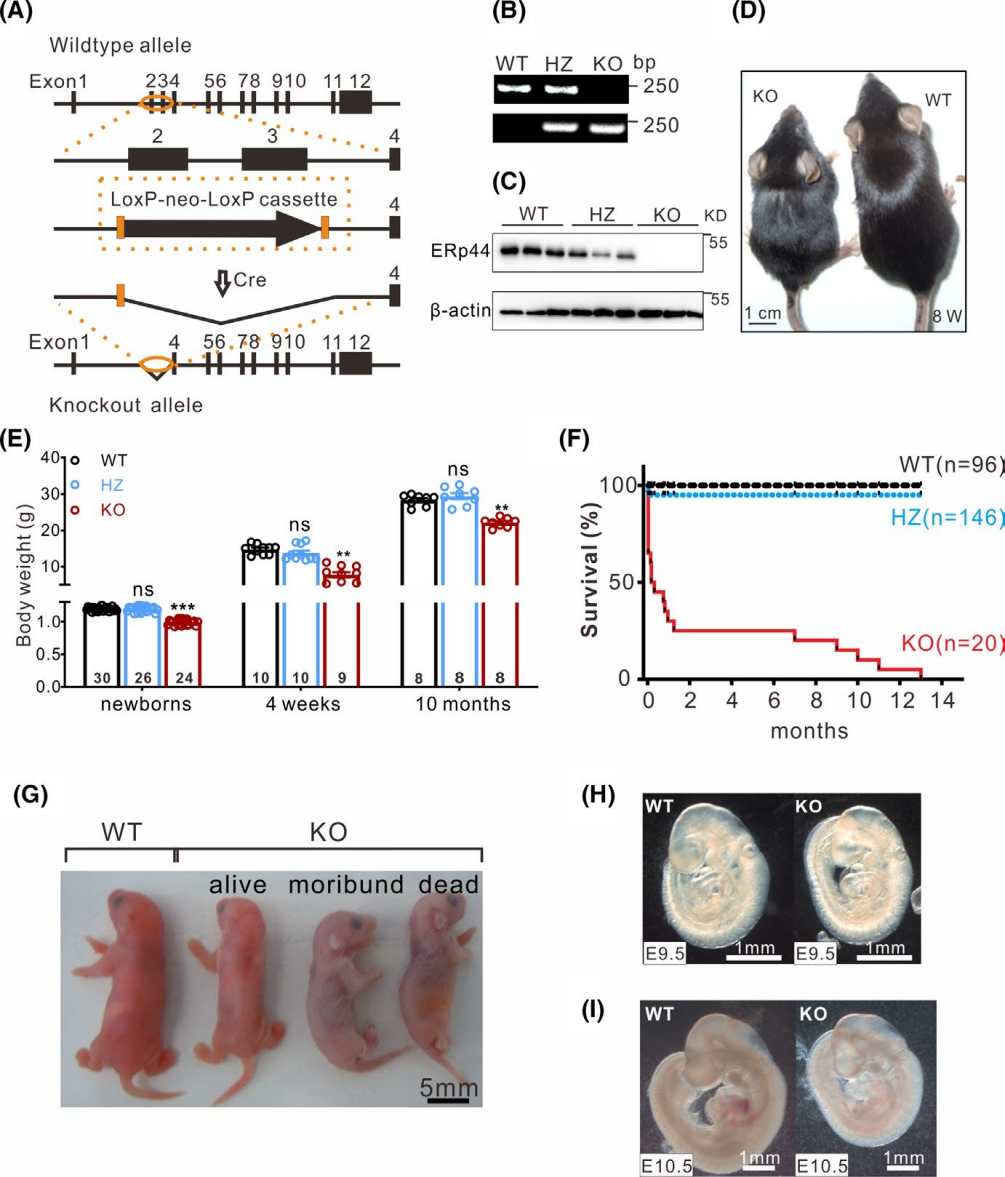
Generation and characterization of ERp44 knockout mice. (A) Strategy used to generate conventional ERp44 knockout mice. (B) Genotyping of WT, heterozygote (HZ) and KO mice. (C) Western blot detection of ERp44 expression in newborn WT, HZ and KO hearts. (D) Photograph of adult WT and KO mice (8 weeks). (E) Body weights of WT, HZ and KO mice of different ages. The statistical data are represented as means ± s. ^**^ *p* < 0.01; ^***^ *p* < 0.001. (F) Survival curve of WT (*n* = 96), HZ (*n* = 146) and KO (*n* = 20) mice from the newborn stage to adulthood. (G–I) Photographs of WT and KO mice at postnatal day (G), E9.5 (H) and E10.5 (I)

To reveal the role of ERp44 in vivo, we generated knockout mice by disrupting the ERp44 gene in embryonic stem cells (ESCs) by homologous recombination (Figure 1A). A genotyping study indicated that exon 2 and exon 3 were successfully deleted (Figure 1B), and ERp44 expression was undetectable in knockout mice, whereas it was observed in WT mice via Western blot (Figure 1C).

### 3.2 ERp44 deficiency leads to perinatal embryonic lethality

To assess the effect of ERp44 deletion in mice, we carefully examined the phenotypes of mice. Specifically, ERp44 KO mice were smaller (Figure 1D and G) and weighed significantly less (Figure 1E) than their littermate controls, and this difference persisted into adulthood. Because the mice were crossed with heterozygotes, the birth rate of KO mice was 21.2% (31/146), slightly lower than the expected Mendelian rate (25%). Most KO mice died within 24 h after birth and exhibited marked cyanosis, and only a few (15% of all KO mice identified after birth) survived to adulthood (Figure 1F). Newborn KO mice initially exhibited normal breathing but turned pale (Figure 1G) and showed symptoms of tachypnoea within a few hours (data not shown), suggesting abnormal cardiopulmonary function. By E9.5, the sizes of KO and WT littermate embryos were similar, but the development of KO embryos was retarded starting at E10.5 (Figure 1H and I).

### 3.3 Embryos deficient in ERp44 exhibit cardiac defects

**FIGURE 2:**
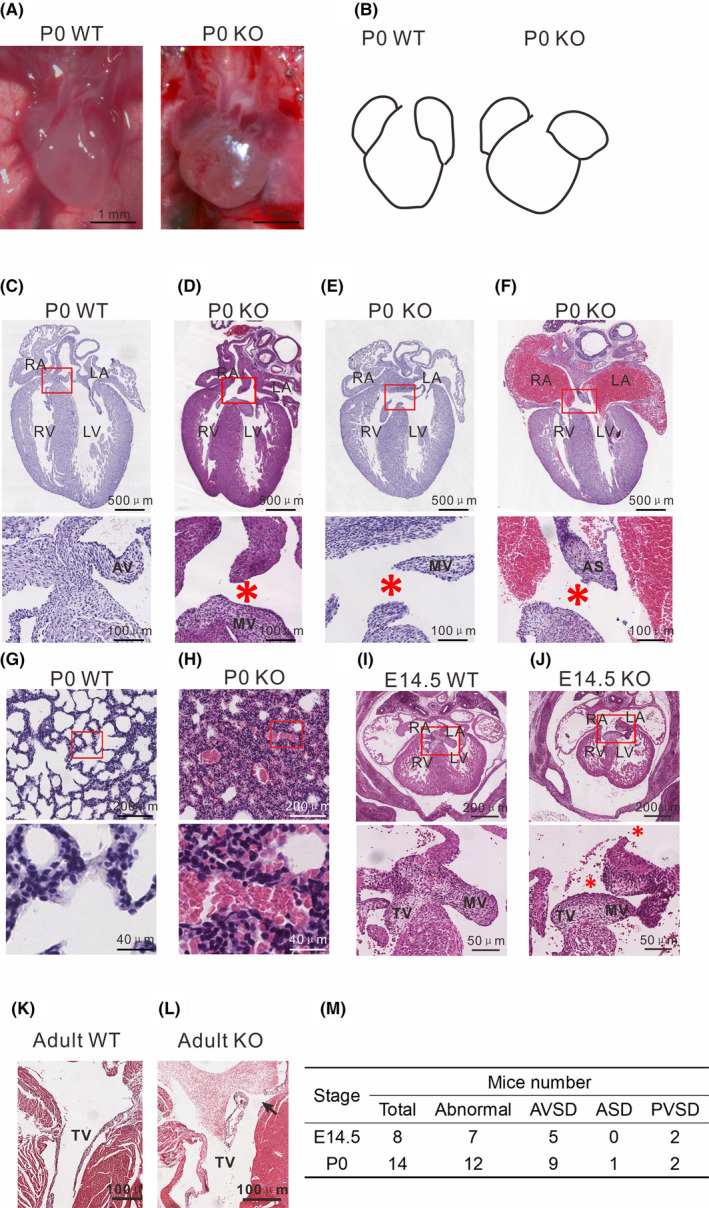
ERp44 deletion causes septal defects in the heart. (A) Photographs of the hearts of WT and KO newborn mice. (B) Schematic map of (A). (C–F) H&E staining of WT (C) and KO (D–F) hearts within 24 h of P0 hearts. Asterisks indicate a defective septum. (D) Primary aortic septal defects (PASDs). (E) Perimembranous ventricular septal defects (PVSDs). (F) Atrioventricular septal defects (AVSDs). (G–H) Pulmonary congestion and oedema in neonatal WT (G) and KO (H) mice. (I–J) Transverse sections of WT (I) and KO (J) hearts at E14.5. (K–L) Transverse sections of adult WT (K) and KO (L) hearts. (M) Frequency of cardiovascular abnormalities found in KO mice at E14.5 and P0. RA, right atrium; LA, left atrium; RV, right ventricle; LV, left ventricle; AV, aortic valve; MV, mitral valve; AS, atrial septum

The observed retarded development of the body and high mortality of ERp44 KO mice after birth suggested that cardiac function is developmentally impaired in these mice. To test this hypothesis, we examined the heart under a stereoscope and found that the hearts of newborn KO mice were biventricularly enlarged and exhibited dilated atria (Figure 2A and F). Histological analysis of serial sections of the organs of newborn mice stained with haematoxylin and eosin (H&E) demonstrated a high incidence of valve defects in the hearts of newborn KO mice compared with WT mice (Figure 2 C‐F and M), including atrioventricular septal defects (AVSDs), primary aortic septal defects (PASDs) and perimembranous ventricular septal defects (PVSDs) (Figure 2D and E). Notably, the muscular septum was unaffected in all KO mice. Pulmonary congestion with focal alveolar oedema was also observed in the lungs of KO mice (Figure 2 G and H), indicating that the pulmonary blood circulation was increased due to a significant left‐to‐right shunt of the heart. The heart defects of ERp44 KO mice were further confirmed by injecting methylene blue into the left ventricle of WT and KO newborn hearts in vivo under a stereoscope. The dye first appeared in the aortic arch in the WT heart, whereas it simultaneously appeared in the right ventricle, right atrium and aortic arch in KO mouse hearts (Figure S1). We further examined serial sections of the hearts at E14.5‐E18.5, when the ventricular septum and primary atrial septum should have been completely closed.^34^ Similar to newborn mice, atrioventricular septation dysplasia and dysplastic valve leaflets were observed in E14.5 ERp44 KO hearts (Figure 2I, J and M). We also observed a slightly dysplastic valve in a survived adult ERp44 KO mouse (Figure 2K and L). These results indicate that ERp44 deletion in mice leads to severe heart defects.

### 3.4 ERp44 deficiency impairs endocardial cushion cell proliferation

**FIGURE 3:**
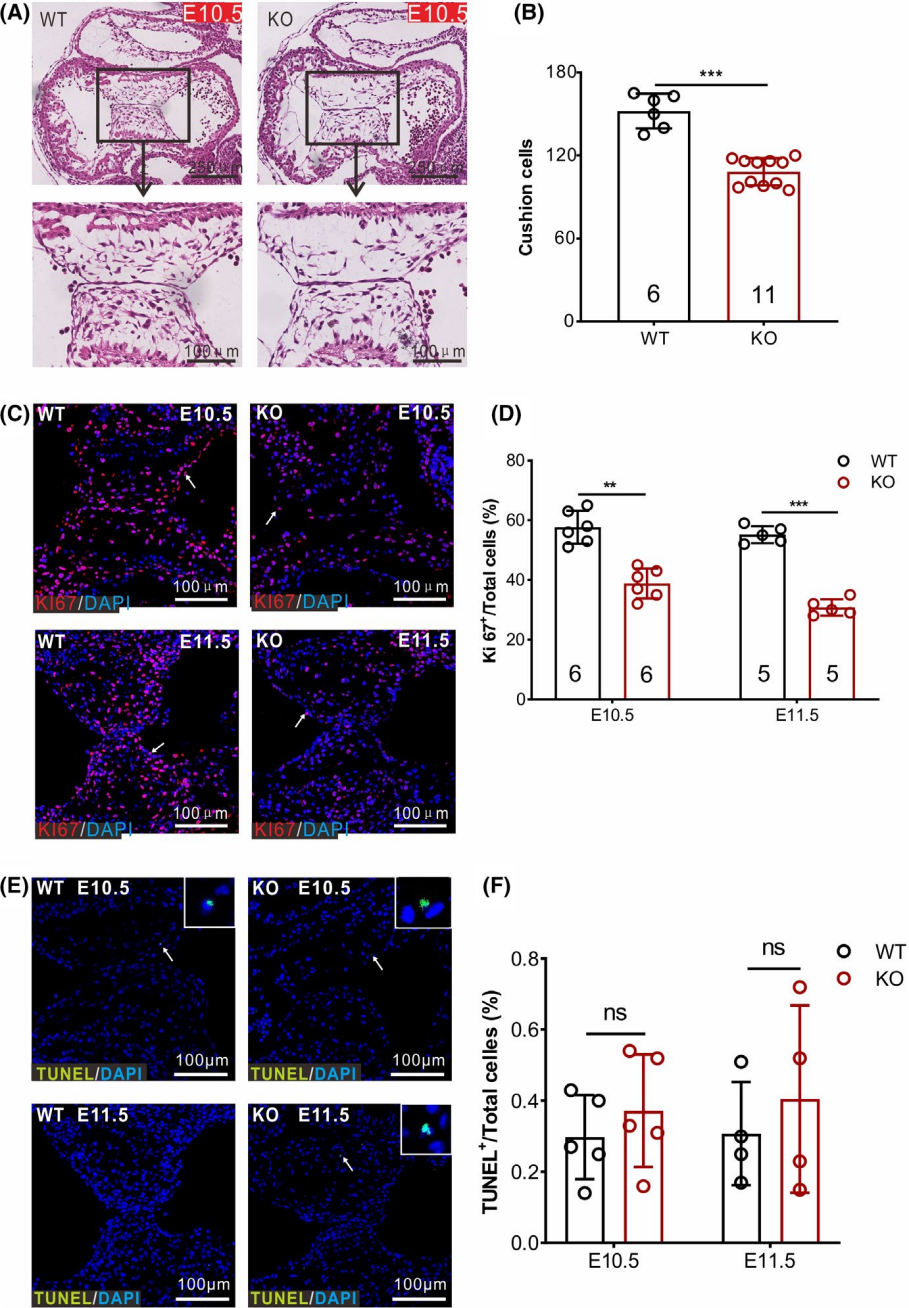
Dysplasia of the endocardial cushion in ERp44 knockout embryos. (A) Histological examination of the atrioventricular canals in WT and KO embryos at E10.5. (B) Quantitation of total endocardial cushion cells. (C and E) Immunostaining of the proliferation marker Ki67 (C) and apoptosis marker TUNEL (E) in WT and KO embryos at E10.5 to E11.5, respectively. (D and F) Quantitation of positive signals (arrows pointed) in Ki67 (D) and TUNEL (F) staining. Arabic numerals in columns represent the experiment number for each genotype. All statistical data are represented as means ± s. ^**^ *p* < 0.01; ^***^ *p* < 0.001

Atrioventricular cushions (AVCs) give rise to atrioventricular septation and valves via a series of complex cellular processes.^5^ We hypothesized that the heart defects observed in ERp44 KO mice were due to the abnormal development of AVCs. To test this hypothesis, we histologically analysed both WT and KO embryo hearts at E10.5 and E11.5. The H&E staining of hearts showed fewer cushion cells in the AVCs of KO mice compared with WT mice (Figure 3A and B). To explore the reason for this decrease, we examined the proliferation and apoptosis of cushion cells with Ki67 (CST, Cat No. 9449) and TUNEL assays (Abcam, Cat No. ab66110), respectively. Compared with WT mice, the number of Ki67‐positive cells was significantly reduced in the AVCs of KO mice (Figure 3C and D), whereas the signs of apoptosis were similar in WT and KO AVCs at E10.5 and E11.5 (Figure 3E and F). Because impairments of the extracellular matrix (ECM) in cardiac jelly result in the abnormal invasion of endocardial‐derived mesenchymal cells,^35^ Alcian blue staining was performed at E9.5, which did not show significant differences in acid glycosaminoglycans in the cardiac jelly between WT and KO AVCs (Figure S2). These results indicate that the hypoplastic AVCs in ERp44 KO mice are primarily due to a proliferative defect of cushion cells.

### 3.5 ERp44 is required for endocardial‐mesenchymal transition (EndMT)

**FIGURE 4:**
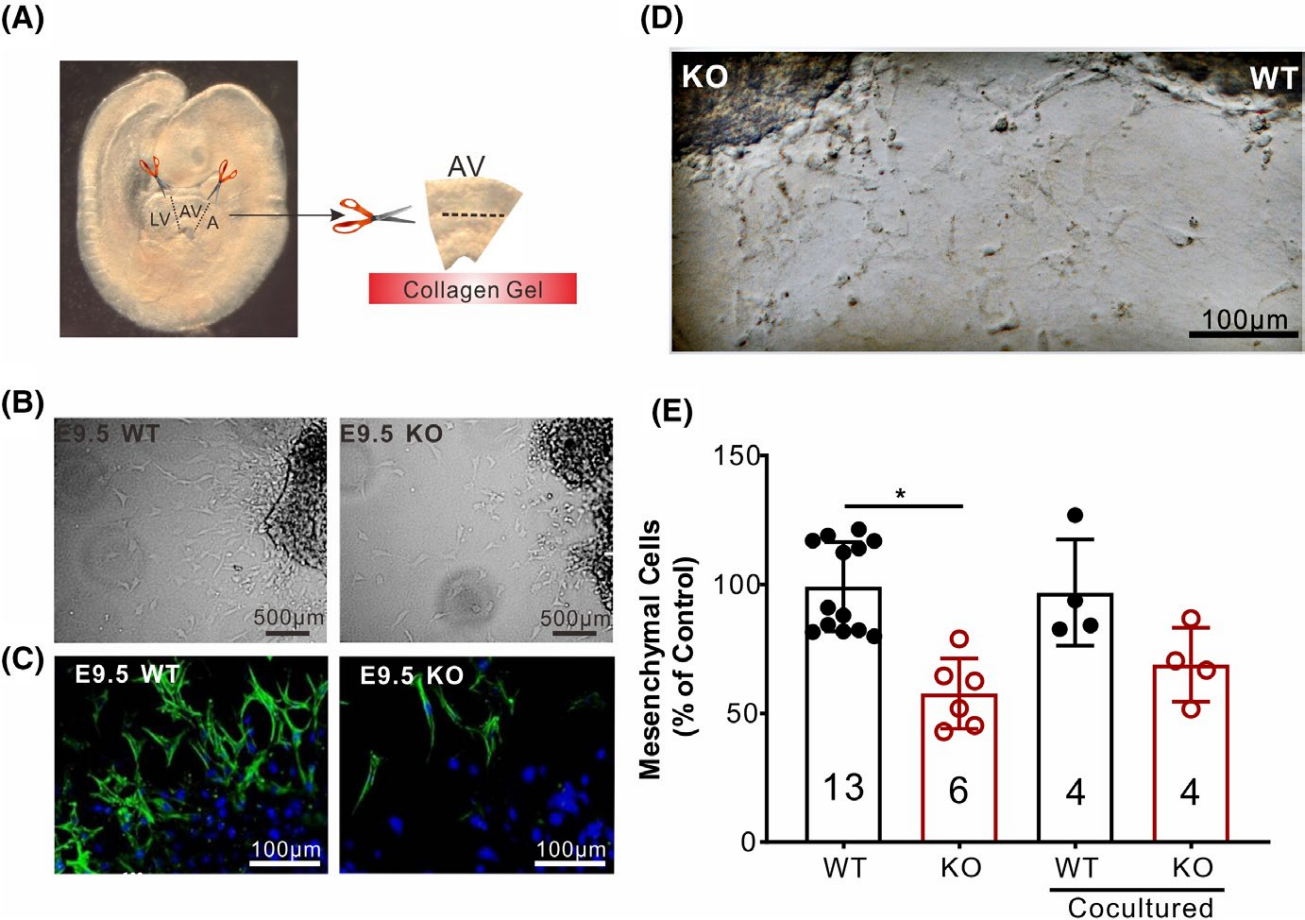
ERp44 is required for EndMT. (A) Schematic diagram to describe the culture of endocardial cushion in vitro. (B) Outgrowths of mesenchymal cells 48 h after endocardial cushion explantation from WT or KO E9.5 embryos as observed by light‐field microscopy. (C) Immunostaining with antibody for the mesenchymal cell marker (αSMA) and for nuclei with DAPI from E9.5 explants. (D) EndMT of adjacently cultured WT and KO cushion explants in E9.5 for 48 h. (E) Statistics of endocardial cushion explantation trial in (B) and (D). The statistical data are represented as means ± s. ^*^ *p* < 0.05

EndMT is a major cellular process during the development of AVCs.^36^ To test whether EndMT is affected in ERp44 KO AVCs, we employed an in vitro assay modified from Feng et al.^30^ Briefly, AVCs from WT and KO E9.5 were explanted (Figure 4A) and cultured on glass plates coated with rat tail collagen gel. After 48 h, large numbers of mesenchymal cells had migrated from the WT explants to the surrounding collagen and exhibited an elongated or spindle‐like shape. In contrast, only a few mesenchymal cells were observed around KO explants, and some exhibited an epithelioid‐like shape, but the cells from KO AVCs had a similar migratory ability (Figure 4B). To confirm our observation, mesenchymal cells were marked with α‐SMA antibody and counted, which showed that WT AVCs contained twice as many mesenchymal cells as KO AVCs (Figure 4C and E). Also, we detected the protein of Aggrecan and Versican^37^ that deeply involved in EndMT, results indicated both of them have a significant decrease in the AVCs of KO mice compared with WT. These results suggested that ERp44 deletion impairs the transition of mesenchymal cells but invalidly affected their morphology and migration.

Furthermore, we investigated whether secreted factors were responsible for the EndMT dysfunction by co‐culture trial of WT and KO endocardial cushion in vitro. The atrioventricular septa of WT and KO mice at E9.5 were isolated, respectively, and they were adjacently co‐cultured in a Petri dish coated with rat tail collagen for 48h. Micrographs showed that plenty of spindle‐shaped mesenchymal cells migrated from the atrioventricular septum. Co‐culture improved the mesenchymal cell migration of KO mice compared with single cultivation (Figure 4D). Also, the mesenchymal migration of WT mice remained unchanged between co‐culture and single cultivation (Figure 4E). These indicated that co‐culture brought out compensation to the mesenchymal cell migration of heart atrioventricular septum in KO mice. Hence, we deduced that the lack of some secreted factors, not excessive secretion of inhibitory factors, was responsible for the above phenotype.

### 3.6 Myocardial loss of ERp44 is the primary cause of heart defects

To further assess the autonomous contribution of cells observed in ERp44 knockout mice, ERp44 conditional knockout mice were generated using the CRISPR/Cas9 technique. Briefly, two loxP sequences were inserted between *ERp44* exon 2 in C57BL/6 mice (Figure S3A), and the *ERp44* floxed allele was inactivated in myocardial cells by crossing the mice with *cTNT*‐*Cre* mice. cTNT‐Cre delivers Cre within the myocardium after E7.5.^38^ The hearts and other organs of *cTNT*‐*Cre* ^+/−^; *ERp44^fl^* ^/+^ mice were collected, and the level of ERp44 mRNA was measured by RT‐PCR to confirm the deletion. *ERp44* showed the shortened mRNA mutant band lacking exon 2 (73 bp) in the heart but not in other tissues (Figure S3B).

**FIGURE 5:**
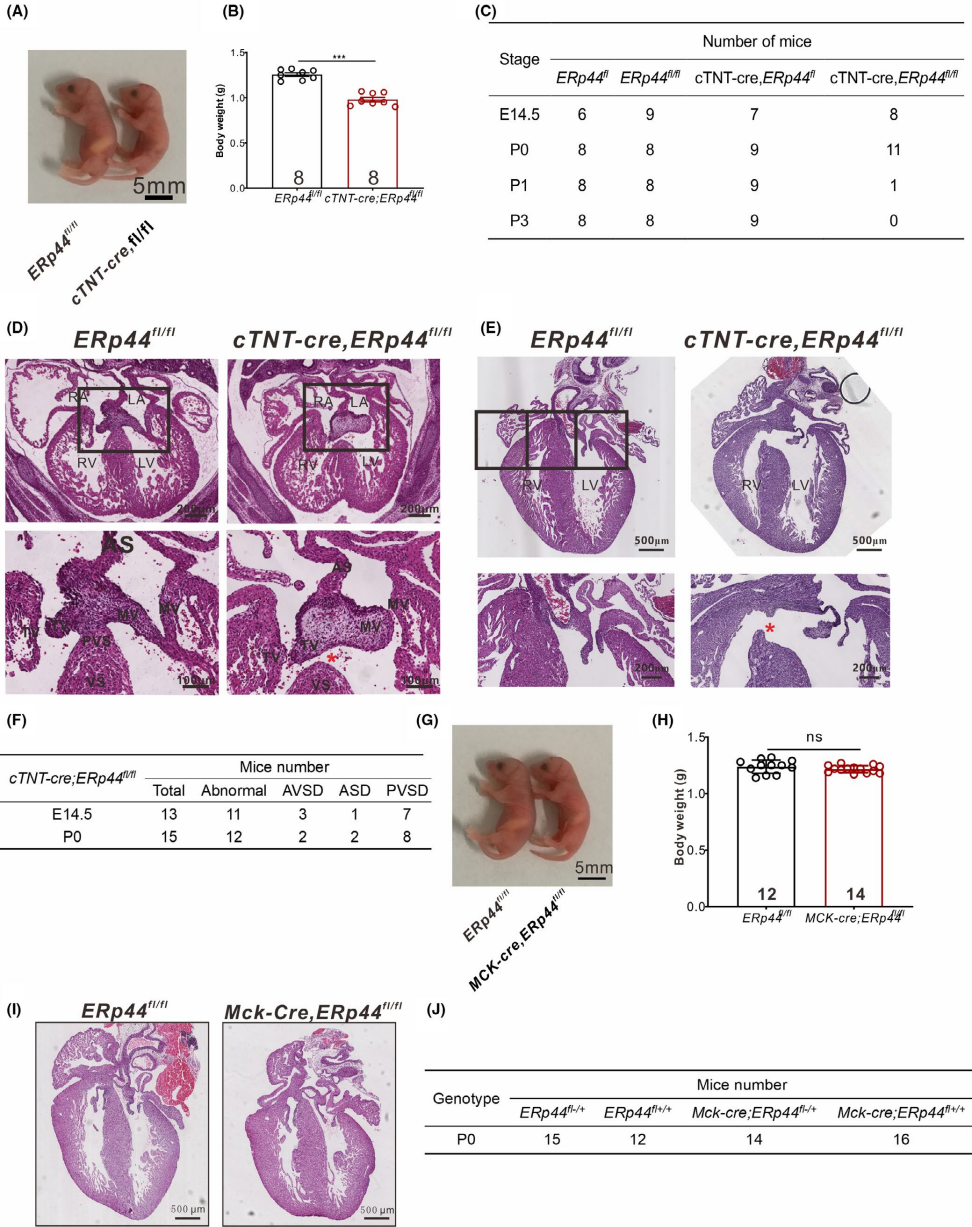
Myocardial‐specific deletion of ERp44 leads to AV cushion defects. (A) Photograph of *ERp44^fl^* ^/^ *^fl^* and *cTNT*‐*Cre* ^+/−^; *ERp44^fl^* ^/^ *^fl^* newborn mice. (B) Quantitation of *ERp44^fl^* ^/^ *^fl^* and *cTNT*‐*Cre* ^+/−^; *ERp44^fl^* ^/^ *^fl^* body weight. All statistical data are represented as means ± s. ^***^ *p* < 0.001. (C) Genotyping results of litters at different stages. (D–E) H&E staining of *ERp44^fl^* ^/^ *^fl^* and *cTNT*‐*Cre* ^+/−^; *ERp44^fl^* ^/^ *^fl^* at E14.5 (D) and P0 (E). Asterisks indicate defective septum. (F) Frequency of cardiovascular abnormalities found in KO mice at E14.5 and P0. (G) Photograph of *ERp44^fl^* ^/^ *^fl^* and *Mck*‐*Cre* ^+/−^; *ERp44^fl^* ^/^ *^fl^* newborn mice. (H) Quantitation of *ERp44^fl^* ^/^ *^fl^* and *Mck*‐*Cre* ^+/−^; *ERp44^fl^* ^/^ *^fl^* body weight. (I) H&E staining of *ERp44^fl^* ^/^ *^fl^* and *Mck*‐*Cre* ^+/−^; *ERp44^fl^* ^/^ *^fl^* at P0. (J) Genotyping results of litters at P0

Because *cTNT*‐*Cre* ^+/−^; *ERp44^fl^* ^/+^ mice were viable and normal, we crossed them with *ERp44^fl^* ^/^ *^fl^ mice* (Figure S3C). Like the conventional KO mice, *cTNT*‐*Cre* ^+/−^; *ERp44^fl^* ^/^ *^fl^* mice were smaller (Figure 5A and B) and exhibited foetal lethality; that is, most mice died at birth (Figure 5C). Histological analysis of *cTNT*‐*Cre* ^+/−^; *ERp44^fl^* ^/^ *^fl^* embryo hearts at E14.5 and hearts within 24 h of birth (P0) showed AVSDs, ASD and PVSDs (Figure 5D–F), which phenocopied the conventional ERp44 KO mice. These results indicate that the myocardial loss of ERp44 is the primary cause of defects.

To validate that myocardial loss of ERp44 is important in AVC development, we crossbred *ERp44^fl^* mice to another muscle‐specific Cre transgenic line *Mck*‐*Cre*. *Mck*‐*Cre* delivers Cre within the myocardium from E13^39^ when AVC development terminates the valve remodelling processes.^40^ *We used Mck*‐*Cre*; *ERp44 ^fl^* ^+^ to cross with *ERp44 ^fl^* ^/^ *^fl^*. The *Mck*‐*Cre*; *ERp44 ^fl^* ^/^ *^fl^* embryos grew normal (Figure 5G and H) and did not show obvious congenital heart defects (Figure 5I), and the birth rate was normal (Figure 5J).

### 3.7 ERp44 KO suppresses VEGFA secretion

**FIGURE 6:**
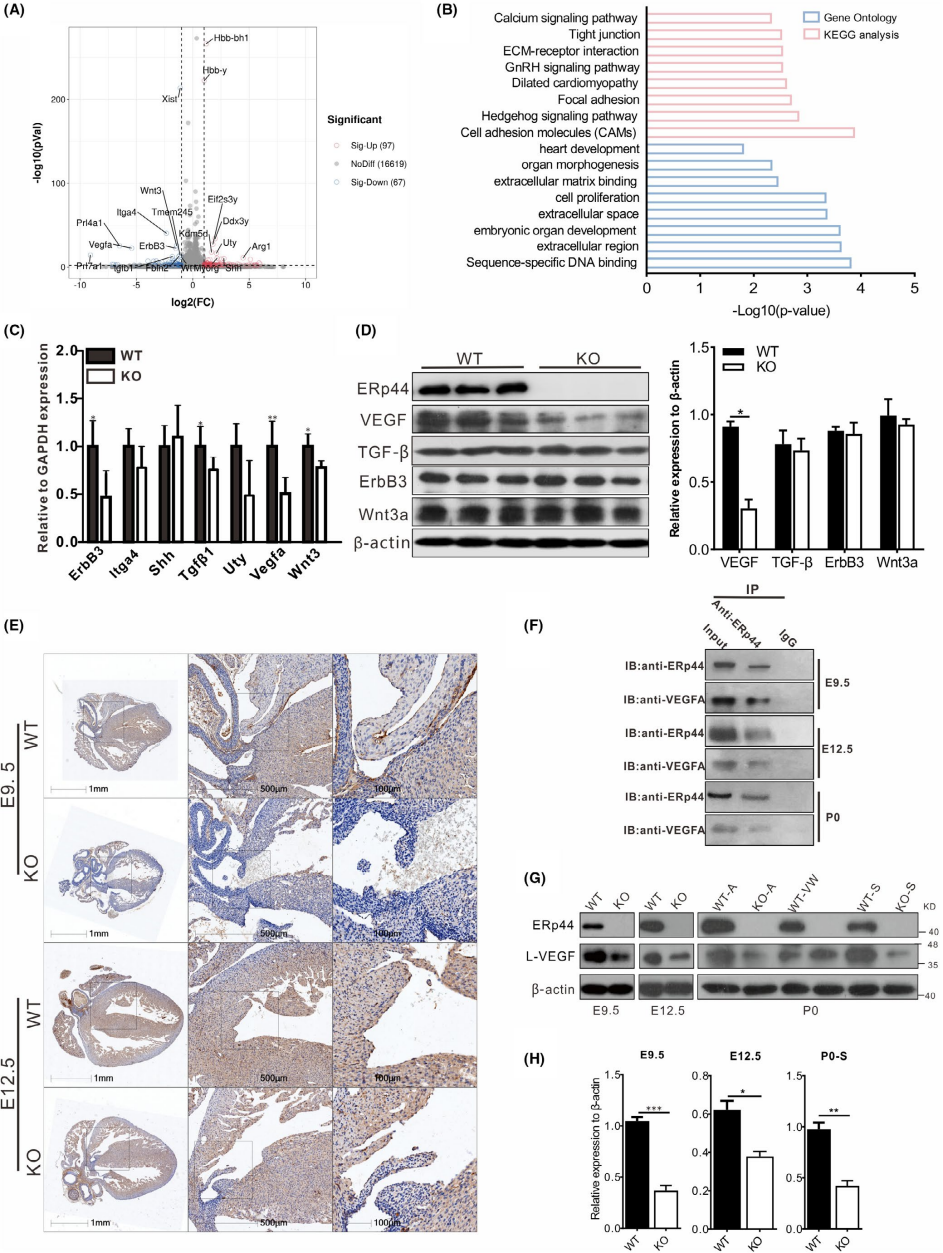
ERp44 KO suppresses VEGFA expression. (A) Volcano description of differential genes (KO vs WT). Sig‐up, significant upregulation; NoDiff, no difference; Sig‐Down, significant downregulation. Top 20 differently expressed genes are listed in the diagram. (B) GO and KEGG analyses depicted the functional classification and pathways of the differentially expressed genes. (C) Real‐time PCR analysis of EndMT‐related genes selected from (A). (D) Western blot detection of multiple crucial proteins that affect the development of mouse endocardial cushion targeting WT and KO AVC tissue in E9.5. β‐Actin was used as an internal reference. (E) Immunohistochemical analysis of VEGF expression in E9.5 and E12.5. (F) Quantification of protein expression in (D). (G) VEGF detection of WT or KO heart in E9.5, E12.5 and P0. A, atrium; VW, ventricular wall; S, septum. (H) Quantification of protein expression in (C). In particular, the atrium, ventricular wall and septum of neonatal heart tissue were separated as previous description.^59^ All statistical data are represented as means ± s. ^*^ *p* < 0.05, ^**^ *p* < 0.01 and ^***^ *p* < 0.001

Multiple signal pathways affect the development of mouse endocardial cushions, such as TGF‐β/BMP, Notch, ErbB, Wnt/catenin and VEGF.^41^, ^42^, ^43^, ^44^ Transcriptome assay and qPCR revealed multiple EndMT‐related genes, such as *ErbB3*, *Itga4*, *Shh*, *Tgfβ1*, *Uty*, *Vegfa* and *Wnt3*, were significantly decreased (Figure 6A and C). The bioinformatic analysis also depicted that differential genes were mainly enriched in these biological processes and pathways related to EndMT, such as extracellular matrix binding, cell proliferation, extracellular region, hedgehog signalling pathway and ECM‐receptor interaction (Figure 6B). At the protein level, only VEGF expression was significantly reduced in KO mice compared with WT (Figure 6D). We next investigated the expression of VEGF at different development stages of the heart. Immunohistochemical trial discovered VEGF had a significant decrease in the defective atrioventricular septum (Figure 6E). Co‐IP trial showed that ERp44 interacted with VEGFA at different stages of heart development (Figure 6F). Western blot assay also indicated VEGF was all markedly decreased in E9.5, E12.5 and the P0 septal tissues of KO mouse hearts compared with WT (Figure 6G and H).

**FIGURE 7:**
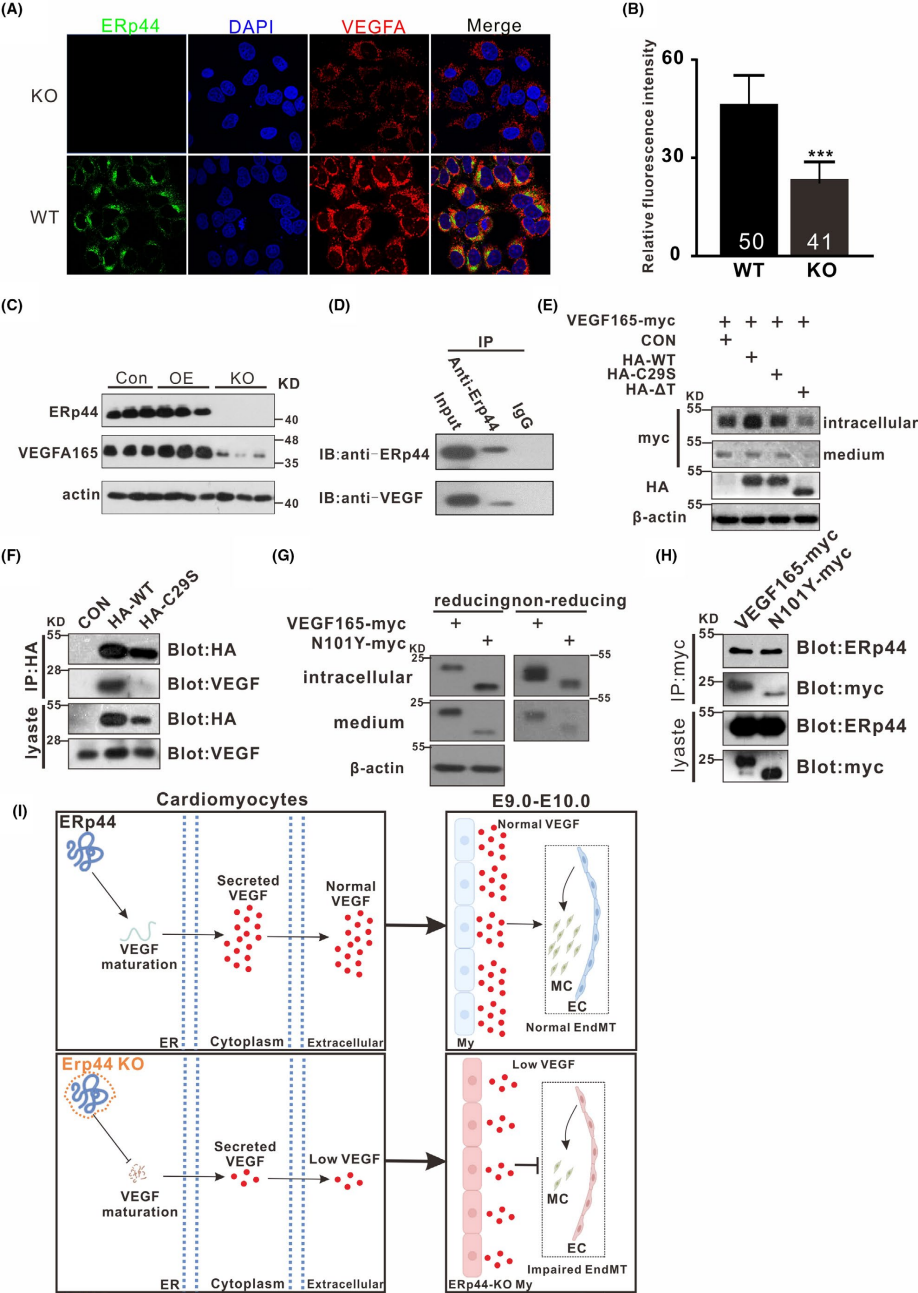
ERp44 regulates the maturation and secretion of VEGF. (A) Micrographs described the expression of VEGFA in WT and ERp44 KO H9C2 cells. The cells were stained with mouse anti‐ERp44 (red) and rabbit anti‐VEGF (green) antibody. The nuclei were stained with DAPI (blue). (B) Relative fluorescence intensity of VEGF in (A). 50 (WT) and 41(KO) fields were taken into consideration. The statistical data are represented as means ± s. ^***^ *p* < 0.001. (C) VEGF expression is positively correlated with ERp44 level. OE and KO represent ERp44 overexpression H9C2 and ERp44 knockout H9C2, respectively. (D) Detection of interaction between ERp44 and VEGF in H9C2 by co‐immunoprecipitation assay. (E) Detection of intracellular and extracellular VEGF‐myc expression in HEK‐293T cells stably expressing VEGF‐myc after transfecting with ERp44‐WT and mutants. β‐Actin was used as an internal reference. CON, *pcDNA3*.*1*; VEGF165‐myc, *pcDNA4*‐*hVEGFA165*‐*myc*; HA‐WT, *pcDNA3*.*1*‐*HA*‐*ERp44*; HA‐C29S, *pcDNA3*.*1*‐*HA*‐*C29S* (C29S mutant of ERp44); HA‐△T, *pcDNA3*.*1*‐*HA*‐*△T* (C‐terminal truncation of ERp44. (F) C29 site of ERp44 contributes to the interaction between ERp44 and VEGF in 293T cells. (G) Glycosylation site N101 of VEGF165 affects its maturation and secretion. VEGFA165‐myc, *pcDNA4*‐*hVEGFA165*‐*myc*; N101Y‐myc, *pcDNA4*‐*N101Y*‐*myc* (N101Y mutant of VEGFA165). (H) Glycosylation site N101 of VEGF165 does not contribute to the interaction between ERp44 and VEGF165. (I) ERp44 is required for EndMT during EC development by regulating VEGF secretion. ERp44 interacts with immature VEGF in endoplasmic reticulum to facilitate its correct fold, maturation and extracellular secretion, and is further involved in the normal EndMT process during EC development. Adversely, ERp44 KO directly results in the decreased extracellular VEGF, which terminates the EndMT early and is responsible for EC dysplasia. EC, endocardial cushion; ER, endoplasmic reticulum; My, myocardium; MC, mesenchymal

To further confirm the relationship between ERp44 and VEGF, we investigated their cytoplasmic localization in H9C2 cells. Micrographs (Figure 7A and Figure S4) and Co‐IP (Figure 7D) analysis revealed ERp44 and VEGF have space co‐localization and certain interaction. Then, we investigated whether ERp44 contributes to VEGF retention within cells. Four expression plasmids, HA‐WT, HA‐C29S, HA‐△T and control (pcDNA3.1), were respectively transfected into 293T cells stably expressing VEGF165‐myc for 48h to detect the VEGF‐myc expression in lysate and supernatant. Results revealed ERp44 overexpression promoted the VEGF165 expression in the cytoplasm, but not in supernatant compared with control. ERp44 mutant(C29S) did not affect the intracellular and extracellular VEGF165 expression, while truncated ERp44 (△T) caused the decrease in VEGF165 within cells and supernatant (Figure 7E). Unlike ERp44, the C29S mutant did not co‐precipitate VEGF, suggesting that efficient binding depends on the Cys29 residue of ERp44 (Figure 7F).

Then, we investigated whether glycosylation site N101 of VEGF165 would affect its interaction with ERp44. After transfecting mutant N101 in 293T, Western blot showed VEGF165 monomer decreased and dimer increased within the cells, and both monomer and dimer decreased in the medium (Figure 7G). This suggested N101 was crucial for the maturation and secretion of VEGF. Yet, co‐expression of ERp44 with VEGF165 or mutant N101Y revealed that glycosylation site N101 did not affect the interaction between ERp44 and VEGF165 (Figure 7H). We also detected VEGF expression in ERp44 OE and ERp44 KO H9C2, respectively. Results showed that VEGF increased in OE H9C2 but notably decreased in KO H9C2 (Figure 7C). Besides, CCK‐8 analysis revealed ERp44 KO suppressed cell proliferation, and VEGF overexpression in ERp44 KO H9C2 cells, to some extent, rescued the above process (Figure S5). These data suggest that ERp44 is involved in the trafficking of VEGF protein.

In summary, ERp44 interacts with immature VEGF in endoplasmic reticulum to facilitate its correct fold, maturation and extracellular secretion, and is further involved in the normal EndMT process during EC development. Adversely, ERp44 KO directly results in the decreased extracellular VEGF, which impairs the EndMT and is responsible for the dysplasia EC (Figure 7I).

## 4 DISCUSSION

Here, we report that ERp44 contributes to the development of endocardial cushion by affecting the proliferation of cushion cells and EndMT. Specifically, myocardial ERp44 principally controlled endocardial cushion formation and the deletion of ERp44 resulted in heart defects by directly regulating the secretion of VEGFA from the myocardium. Although Wang et al. firstly discovered ERp44 deletion was responsible for the cardiac dysfunction proved by disordered Ca^2+^ signalling, ER stress and myocardial apoptosis,^25^ they did not clarify the insufficiency of atrioventricular septal in morphological structure at different stages of development.

Mice with congenital heart defects were smaller and exhibited growth retardation and embryonic lethality, although some mice may survive after birth, as previously reported.^45^, ^46^, ^47^ Our findings showed that most KO mice died within the first day of birth, although a few mice survived until adulthood and exhibited slight valve dysplasia and slightly abnormal cardiac morphology. The few surviving adult KO mice did not show significant cardiac hypertrophy on an echocardiogram (data not shown). Wang et al reported that their ERp44 mutant mice died mainly at E12.5,^25^ and their surviving adult KO mice showed cardiac hypertrophy, which contradicted our observations. This difference may be because they inserted a *LacZ* gene into *ERp44* exon 1 as a fusion protein, which may have caused additional embryonic toxicity, whereas we deleted exon 2 and exon 3 from the *ERp44* allele. Using another ERp44 mutant mouse line (deleting exon 2 and exon 3 of *ERp44*), Hisatsune et al obtained similar results to ours.^26^

We found that ERp44 KO mice were predominantly AVSD, with some PVSD and ASD and no apparent defect in the myocardium. These results showed dysplasia in the AV cushion development. The proportion of abnormal KO heart was approximately 85%, which was in line with the lethality rate (about 80%). The fact that CHD phenotype penetrance was lower than 100% was also reported by other studies.^47^, ^48^ Possible explanations for this reason are as follows: 1, the defects were not found by serial section; and 2, the mild defects may heal at late‐developing stages. As no obvious defects were found in KO mice related to OFT cushion development, we did not investigate particularly whether the OFT cushion EndMT was impaired. An explanation of these anomalies is that mesenchymal cells derived from cardiac neural crest cells can migrate in this area to a certain extent.^49^, ^50^

AV cushion morphogenesis proceeds in the time window from E9.5 to E11.5 in mice and has been widely reported to be controlled by signals from both the endocardium and myocardium.^36^, ^51^ After E11.5, heart valve remodelling proceeds.^40^ Using myocardial‐specific Cre mice (*cTNT*‐*Cre* and *Mck*‐*Cre*), we observed phenotypes of *cTNT*‐*Cre*; *ERp44^fl^* ^/^ *^fl^* similar to those observed in conventional knockout mice, while *Mck*‐*Cre*; *ERp44^fl^* ^/^ *^fl^* mice showed no significant phenotype in heart and could grow to adulthood. The explanation of this phenomenon is that *cTNT*‐*Cre* delivers Cre from E7.5 and *Mck*‐*Cre* from E13. These indicate that AV cushion defects are primarily due to hypoplastic EndMT by a loss of myocardial ERp44. Nevertheless, we cannot exclude the role of endocardial ERp44 in our mouse model.

The major signals from the myocardium are BMP2/4, TGF‐β1/2/3, Notch and VEGFA.^38^, ^41^, ^52^ Combining transcription analysis, qPCR and WB, we found that VEGF decreased in E10.5 ERp44 KO AVC tissue at the protein level compared with littermate controls, and the similar phenomenon appeared in different development stages of hearts in KO mice, suggesting that less VEGF was secreted in ERp44 KO AVCs. Ramming et al speculated that VEGF might be directly regulated by ERp44.^53^ Here, we confirmed that ERp44 interacted with VEGFA and positively controlled VEGFA secretion. VEGFA signalling plays a spatiotemporal role in controlling EndMT, and disrupting one VEGFA allele or overexpressing VEGFA causes AV cushion dysplasia.^54^ In the myocardium, the resting calcium level also regulates EndMT. Specifically, an increase in the intracellular calcium concentration can activate calcineurin, which promotes the translocation of NFATc2/3/4 into the nucleus to regulate the expression of a set of genes, including VEGFA.^40^, ^55^ However, we did not observe differences in the resting Ca^2+^ level in isolated cardiomyocytes between KO and WT mice at P0 (data not shown). The importance of ERp44 in the early secretion pathway has been well studied.^16^, ^17^, ^26^, ^56^, ^57^ Several proteins have been found to interact with ERp44, including IgM,^17^ adiponectin^58^ and ERAP1,^26^ and other clients, such as VEGFA, are speculated to bind to ERp44.^53^ Here, we found that ERp44 directly binds to VEGFA, and C29 in ERp44 was important for the binding with VEGFA. Also, the glycosylation site of VEGF N101 was critical for its maturation and secretion but affects less on the interaction between ERp44 and VEGF165.

In conclusion, we discovered a novel and close link between the ER chaperone protein ERp44 and endocardial cushion defects. Specifically, myocardial ERp44 contributes to the development of the endocardial cushion by affecting the proliferation of cushion cells and EndMT processes, and ERp44 directly regulates VEGFA by binding to the C29 site. The information gained from this study will improve our understanding of the mechanisms underlying AV cushion defects and may also provide a potential diagnostic strategy for congenital heart defects in humans.

## 5 ETHICS STATEMENT

All animal experiments were performed following the guidelines of laboratory animal care (NIH publication NO. 85‐23, revised 1996) and with approval from the Institute of Biophysics Committee for Animal Care (Approval No. SYXK2019025).

## CONFLICT OF INTERESTS

The authors declare no competing or financial interests.

## AUTHOR CONTRIBUTIONS

Youkun Bi, Zhiguang Yang, Fengchao Wang, Hong Cai and Guangju Ji conceived and designed the experiments. Youkun Bi, Zhiguang Yang, Meng Jin, Kui Zhai, Jun Wang, Yang Mao, Yang Liu and Huiwen Wang performed the experiments and contributed reagents/materials/analysis tools. Mingqin Ding and Fengchao Wang performed the design of ERp44 KO mice. Youkun Bi, Hong Cai and Guangju Ji wrote the manuscript. All the authors have full access to the data in the study and final responsibility for the decision to submit for publication.

## Supporting information

Fig S1

Fig S2

Fig S3

Fig S4

Fig S5

Supplementary Material

Table S1

Table S2

Table S3

Supplementary Material

## Data Availability

Main data generated or analysed during this study are included in this article, and detailed data are available from the corresponding authors on reasonable request.
