# Supplementary figures and images for: ERp44 is required for endocardial cushion development by regulating VEGFA secretion in myocardium
Source: Cell Prolif. 2022 Jan 28;55(3):e13179. doi: 10.1111/cpr.13179 (PMC8891561; doi:10.1111/cpr.13179)

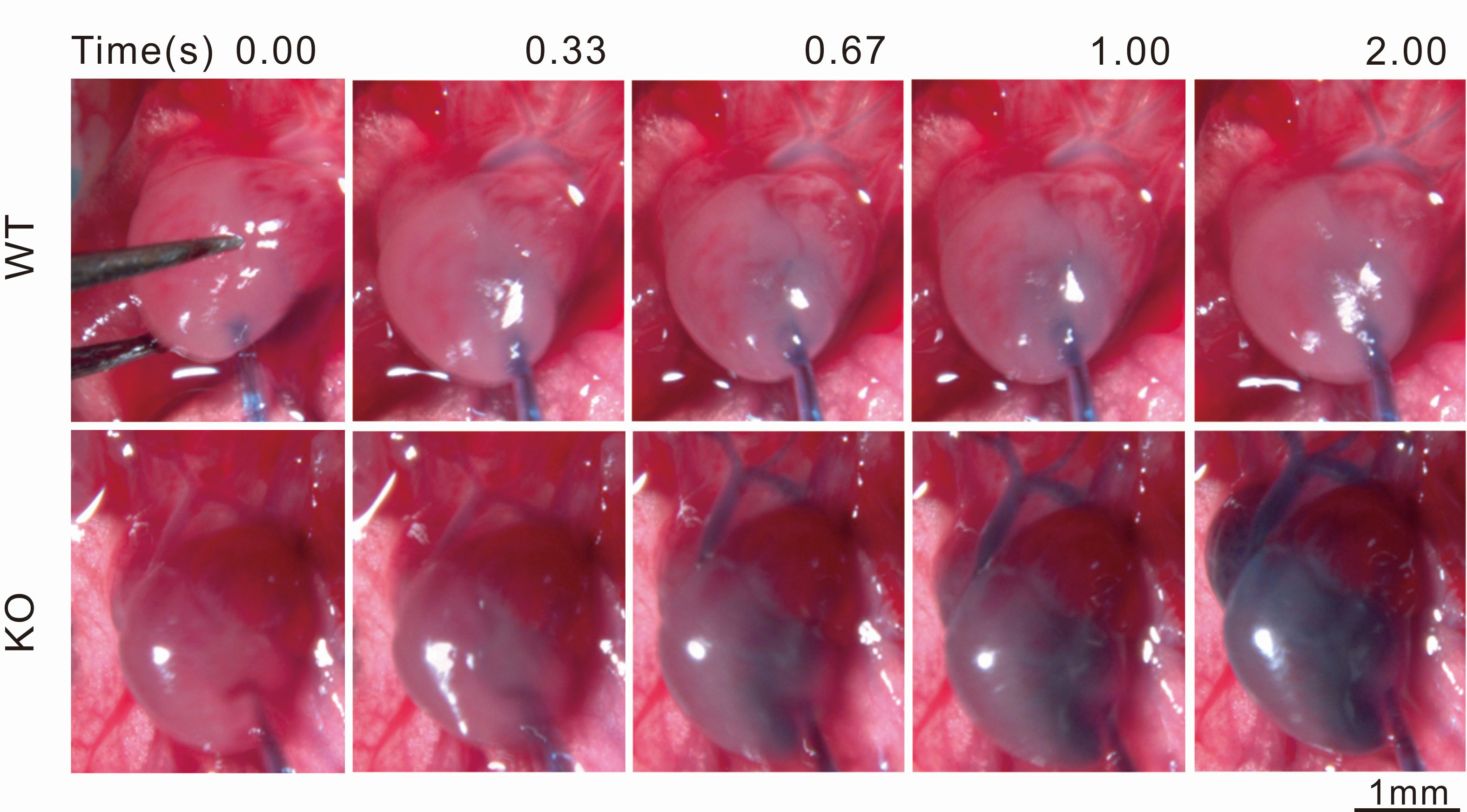

Supplement: Supplementary file 1 — Fig S1 [file CPR-55-e13179-s005.tif]

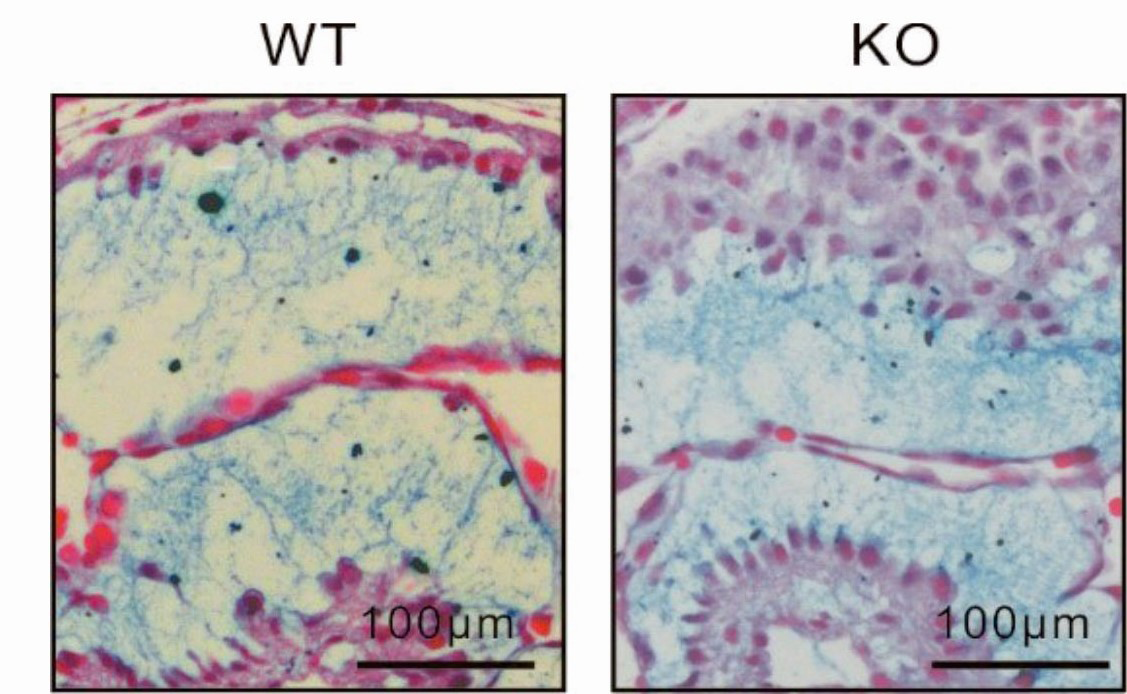

Supplement: Supplementary file 2 — Fig S2 [file CPR-55-e13179-s003.tif]

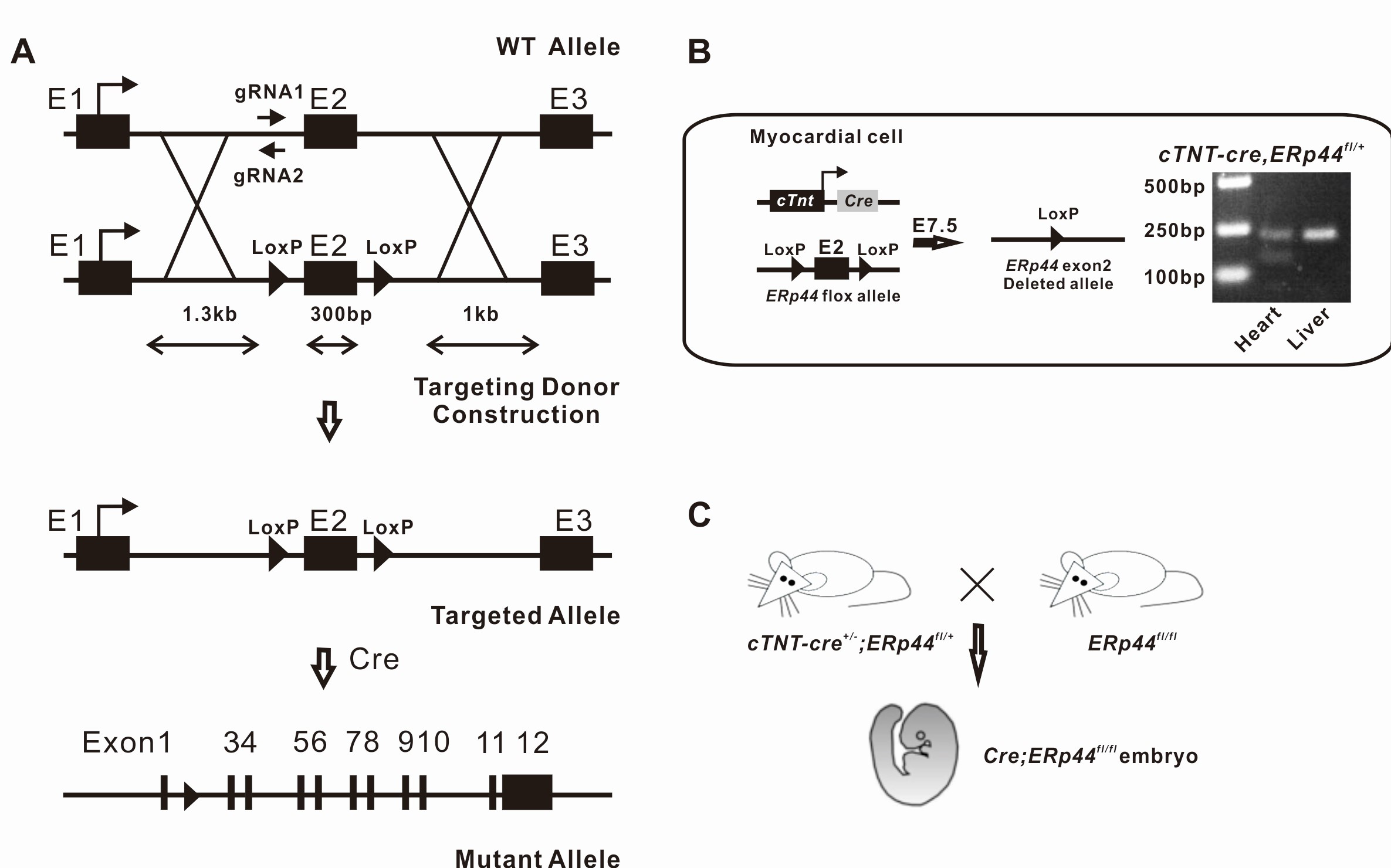

Supplement: Supplementary file 3 — Fig S3 [file CPR-55-e13179-s010.tif]

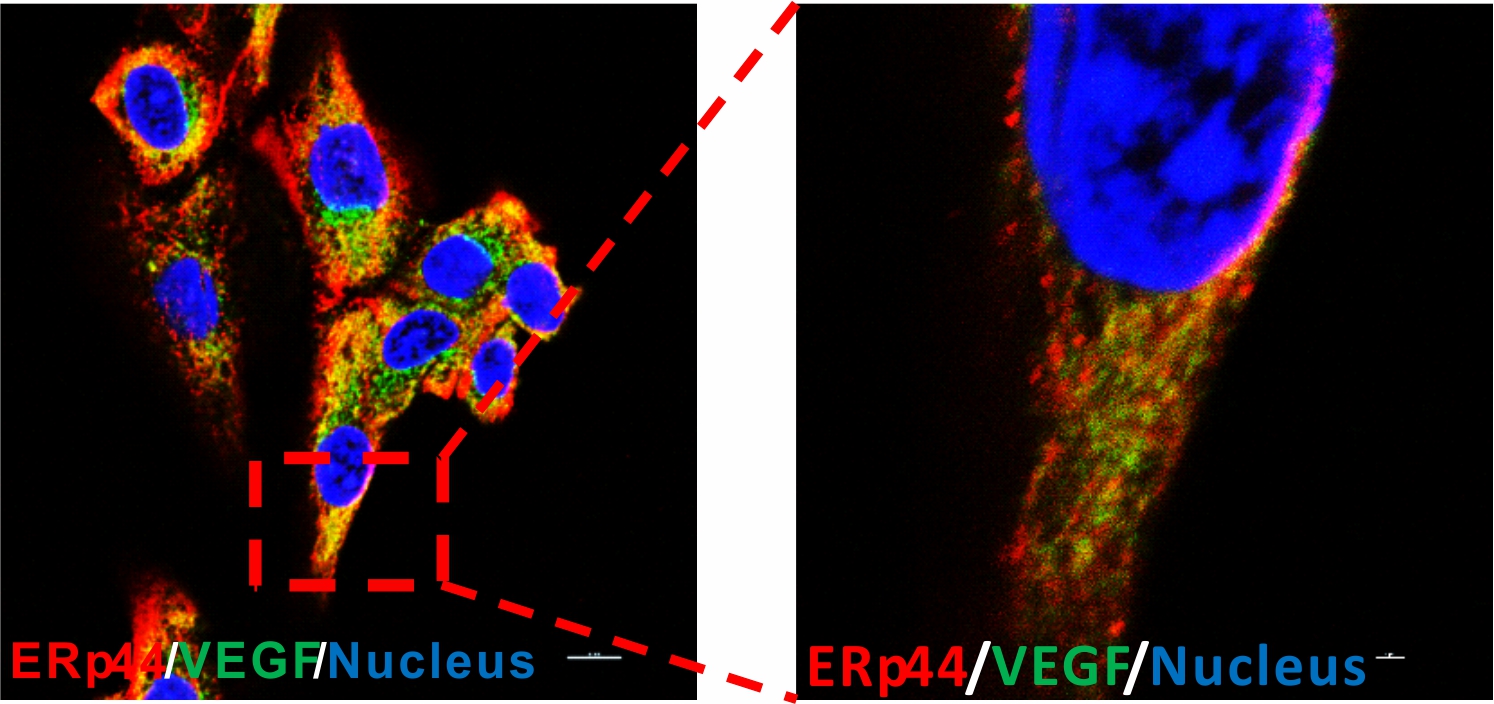

Supplement: Supplementary file 4 — Fig S4 [file CPR-55-e13179-s004.jpg]

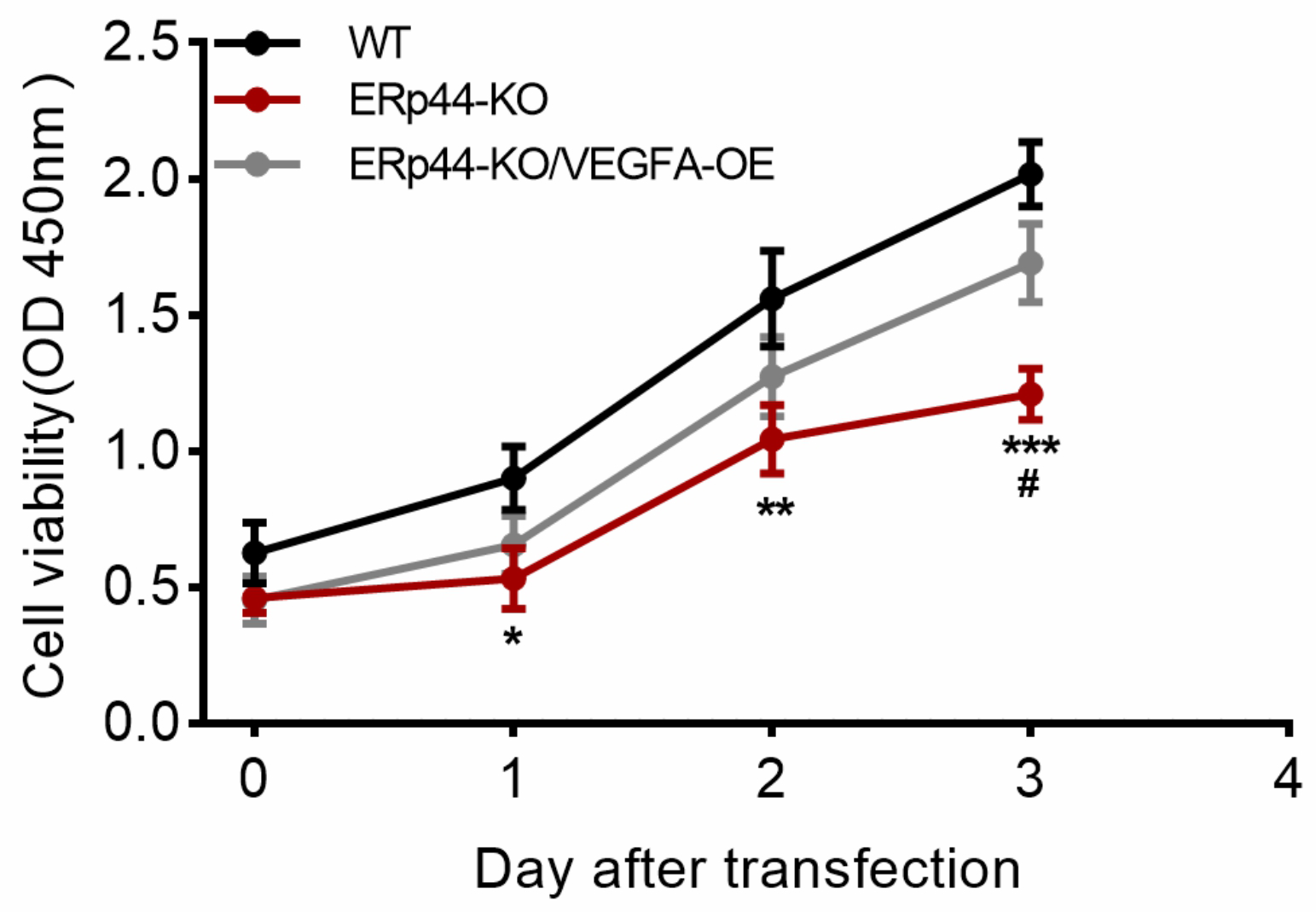

Supplement: Supplementary file 5 — Fig S5 [file CPR-55-e13179-s009.tif]
