# Supplementary material for: ERp44 is required for endocardial cushion development by regulating VEGFA secretion in myocardium
Source: Cell Prolif. 2022 Jan 28;55(3):e13179. doi: 10.1111/cpr.13179 (PMC8891561; doi:10.1111/cpr.13179)
Supplement: Supplementary file 6 — Supplementary Material [file CPR-55-e13179-s007.docx]

**Supplementary materials of**

**ERp44 is Required for Endocardial Cushion Development by Regulating VEGFA Secretion in Myocardium**


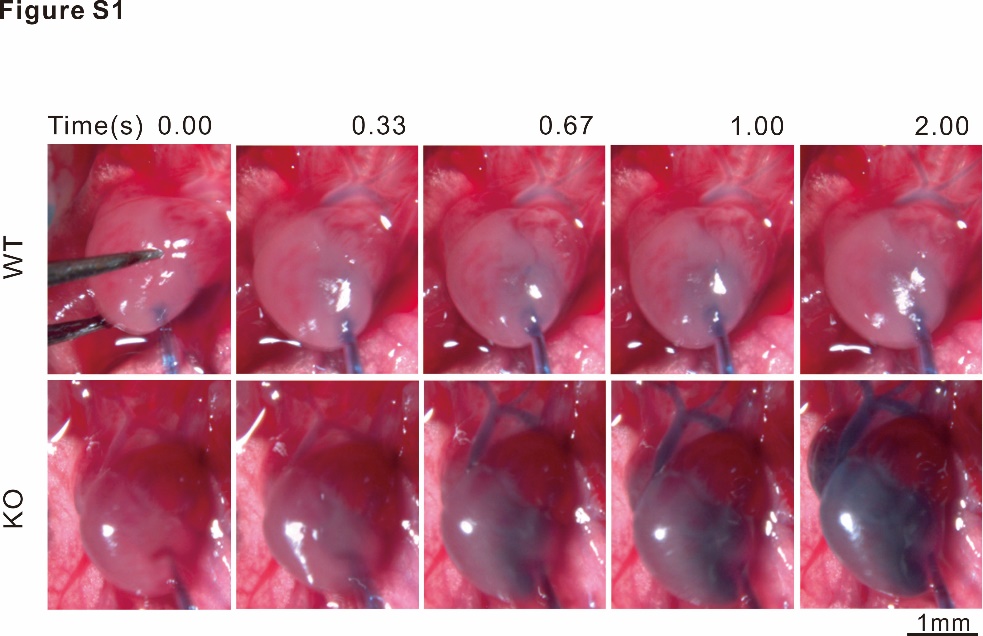
**Supplementary Figures**

**Fig. S 1.** Exploration of hemodynamics in vivo. Methylene blue (1mg/ml) was gently injected into the left ventricle of newborn WT or KO hearts (0.00 s). The dye passed through the aortic arches in both hearts (0.33 s) and filled the right atrium and auricle of KO mice 1.67 s later instead of passing into systemic circulation, similar with WT mice (2.00 s).


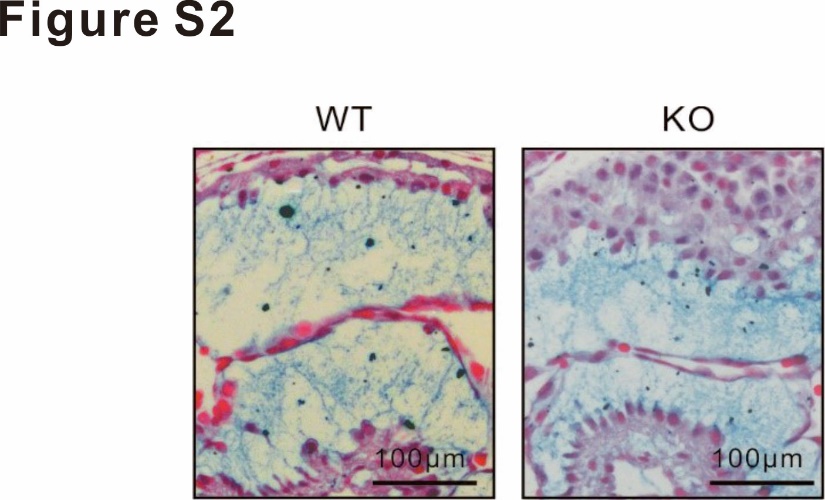


**Fig. S 2.** Alcian blue staining of AVCs in E9.5 WT and KO embryos.


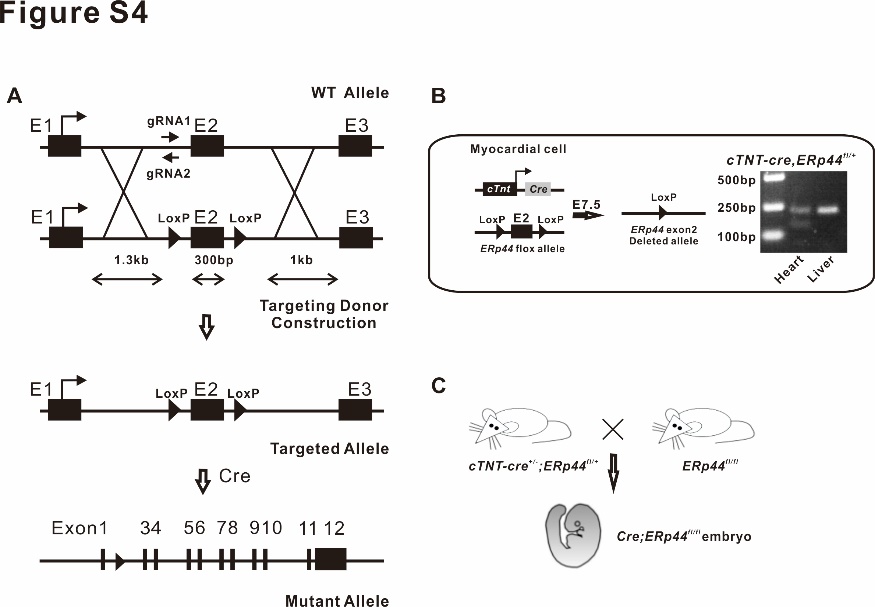
**Fig. S 3.** Strategy used to generate conventional ERp44 knockout mice. (A) gRNA and Cas9 mRNA were injected into C59BL zygotes. The targeting donor was designed to replace exon 2 of the wild-type allele with two flanking loxP sequences (shown LoxP). Two homologous arms were used for homologous repair. (B) Schematic showing the ERp44 deletion in myocardial cells by *cTnt-cre*. RT-PCR showed an additional 73-bp-shorter band in the heart but not liver of *cTnt-cre;Erp44^fl/+^*mice. (C) Schematic showing the crossing strategy. *cTnt-cre^+/-^;Erp44^fl/^* mice were crossed with *Erp44^fl/fl^* mice.


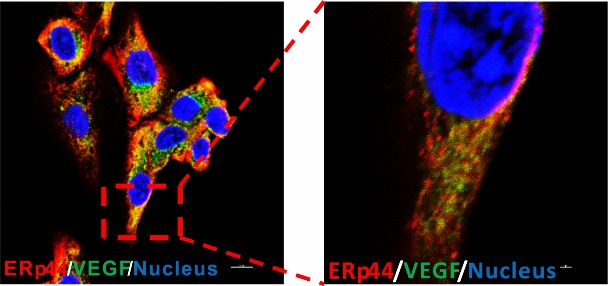


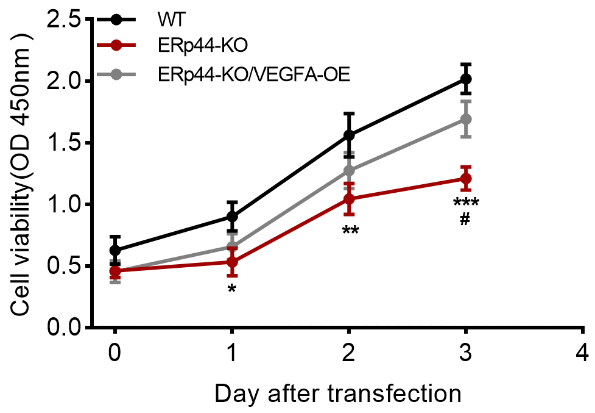
**Fig. S 4.** Micrographs described the colocalization of ERp44 and VEGF in H9C2. The cells were stained with mouse anti-ERp44 (red) and rabbit anti-VEGF (green) antibody. The nuclei were stained with DAPI (blue).

**Fig. S 5. Cell viability measurement.** CCK-8 assay showed ERp44-KO H9C2 cells had lower cell viability than WT. Overexpression VEGF in ERp44-KO H9C2 cells, to some extent, rescued the decreased cell viability. Three independent repetitions were performed. All statistical data are represented as means ± s. **p*<0.05; ^*^*p*<0.01 (ERp44-KO vs WT); ^#^*p*<0.05 (ERp44-KO vs ERp44-KO/VEGFA-OE).

| **Supplementary tables**  **Table S1: Sequence information of genotyping primers.** | |
| --- | --- |
| **Primers** | **Sequence** |
| ERp44-GF1 | TATTCTGAGCCAAAGCACTTCAG |
| ERp44-GR1 | CTCCAGGCTCTGTCTGAGATG |
| ERp44-WT-F | TTACTCTCCGTGCTGCTTGG |
| ERp44-WT-R | GACTTGCATGAGGGGGTACT |
| ERp44-sequence-F | AGGCTCTGTCTGAGATGAATGC |
| ERp44-sequence-R | CAGTAATAAAGTTGGCTCTACCTGTT |
| ERp44-screen-F | ATTTCTATTTTATTGTTCTAATAAATAATGGGA |
| ERp44-screen-R | GACTTCTCCTTCTAAGGGGACTTGC |
| RT-ERp44-exon2-F | CTTTTCTAGCCCGGTCCAGT |
| RT-ERp44-exon2-R | TGACATCAGATGCTTCCTCAA |

| **Table S2. Sequence information of RT-qPCR primers.** | | |
| --- | --- | --- |
| **Gene symbol** | **Forward sequence** | **Reverse sequence** |
| GAPDH | TGGCCTTCCGTGTTCCTAC | GAGTTGCTGTTGAAGTCGCA |
| ErbB3 | AGGCTCATTGCTTCTCCTGCCA | GAAAATGGGCGCATCGAGCACA |
| Itga4 | GCAAAGAGGTCCCAGGCTACAT | CCTGTAATCACGTCAGAAGTCCC |
| Shh | GGATGAGGAAAACACGGGAGCA | TCATCCCAGCCCTCGGTCACT |
| Tgfβ1 | TGATACGCCTGAGTGGCTGTCT | CACAAGAGCAGTGAGCGCTGAA |
| Uty | CAACAGAAGTTCTGAAAGCGTGC | GGAGGATATGGCGAAGTTGGTG |
| Vegfa | CTGCTGTAACGATGAAGCCCTG | GCTGTAGGAAGCTCATCTCTCC |
| Wnt3 | CCGCTCAGCTATGAACAAGCAC | AAGTCGCCAATGGCACGGAAGT |

**Table S3. Sequence information of sgRNA for ERp44 knockout.**

| **Primers** | **Sequence** |
| --- | --- |
| ERp44-sgRNA-F | TTGATGTAAGTTACTTATGG |
| ERp44-sgRNA-R | CCATAAGTAACTTACATCAA |

**Antibody list**

| **Primary Antibody** | **Company (Catalogue NO.)** | **Dilution** |
| --- | --- | --- |
| ERp44 | Santa cruz, Cat^#^sc-393687 | 1:1000 for WB; 1:500 for IF |
| Ki67 | CST, Cat^#^9449 | 1:1000 for IF |
| α-SMA | CST, Cat^#^19245 | 1:100 for IF |
| VEGFA | Abcam, Cat^#^ab52917 | 1:1000 for WB; 1:100 for IHC |
| TGFβ1 | Abcam, Cat^#^ab215715 | 1:1000 for WB |
| ErbB3 | Abcam, Cat^#^ab255607 | 1:1000 for WB |
| Wnt3a | Abcam, Cat^#^ab219412 | 1:1000 for WB |
| β-actin | CST, Cat^#^3700S | 1:1000 for WB |
| HA-tag | CST, Cat^#^2367S | 1:1000 for WB |
| Myc-tag | CST, Cat^#^s2276S | 1:1000 for WB |

| **Secondary Antibody** | **Company (Catalogue NO.)** | **Dilution** |
| --- | --- | --- |
| Anti-rabbit IgG, HRP-coupled Antibody | CST, Cat^#^7074S | 1: 2000 |
| Anti-mouse IgG, HRP-coupled Antibody | CST, Cat^#^7076S | 1: 2000 |
| Goat anti-rabbit IgG(H+L) secondary, Alexa Fluor 488 | ZSGB-BIO, Cat^#^ZF-0511 | 1: 200 |
| Goat anti-rabbit IgG(H+L) secondary, Alexa Fluor 594 | ZSGB-BIO, Cat^#^ZF-0516 | 1: 200 |
| Goat anti-mouse IgG(H+L) secondary, Alexa Fluor 488 | ZSGB-BIO, Cat^#^ ZF-0512 | 1: 200 |
| Goat anti-mouse IgG(H+L) secondary, Alexa Fluor 594 | ZSGB-BIO, Cat^#^ ZF-0513 | 1: 200 |
