# Supplementary material for: ERp44 is required for endocardial cushion development by regulating VEGFA secretion in myocardium
Source: Cell Prolif. 2022 Jan 28;55(3):e13179. doi: 10.1111/cpr.13179 (PMC8891561; doi:10.1111/cpr.13179)
Supplement: Supplementary file 7 — Table S1 [file CPR-55-e13179-s008.docx]

| **Table S1: Sequence information of genotyping primers.** | |
| --- | --- |
| **Primers** | **Sequence** |
| ERp44-GF1 | TATTCTGAGCCAAAGCACTTCAG |
| ERp44-GR1 | CTCCAGGCTCTGTCTGAGATG |
| ERp44-WT-F | TTACTCTCCGTGCTGCTTGG |
| ERp44-WT-R | GACTTGCATGAGGGGGTACT |
| ERp44-sequence-F | AGGCTCTGTCTGAGATGAATGC |
| ERp44-sequence-R | CAGTAATAAAGTTGGCTCTACCTGTT |
| ERp44-screen-F | ATTTCTATTTTATTGTTCTAATAAATAATGGGA |
| ERp44-screen-R | GACTTCTCCTTCTAAGGGGACTTGC |
| RT-ERp44-exon2-F | CTTTTCTAGCCCGGTCCAGT |
| RT-ERp44-exon2-R | TGACATCAGATGCTTCCTCAA |
