# Supplementary material for: ERp44 is required for endocardial cushion development by regulating VEGFA secretion in myocardium
Source: Cell Prolif. 2022 Jan 28;55(3):e13179. doi: 10.1111/cpr.13179 (PMC8891561; doi:10.1111/cpr.13179)
Supplement: Supplementary file 8 — Table S2 [file CPR-55-e13179-s001.docx]

| **Table S2. Sequence information of RT-qPCR primers.** | | |
| --- | --- | --- |
| **Gene symbol** | **Forward sequence** | **Reverse sequence** |
| GAPDH | TGGCCTTCCGTGTTCCTAC | GAGTTGCTGTTGAAGTCGCA |
| ErbB3 | AGGCTCATTGCTTCTCCTGCCA | GAAAATGGGCGCATCGAGCACA |
| Itga4 | GCAAAGAGGTCCCAGGCTACAT | CCTGTAATCACGTCAGAAGTCCC |
| Shh | GGATGAGGAAAACACGGGAGCA | TCATCCCAGCCCTCGGTCACT |
| Tgfβ1 | TGATACGCCTGAGTGGCTGTCT | CACAAGAGCAGTGAGCGCTGAA |
| Uty | CAACAGAAGTTCTGAAAGCGTGC | GGAGGATATGGCGAAGTTGGTG |
| Vegfa | CTGCTGTAACGATGAAGCCCTG | GCTGTAGGAAGCTCATCTCTCC |
| Wnt3 | CCGCTCAGCTATGAACAAGCAC | AAGTCGCCAATGGCACGGAAGT |
