# Supplementary material for: ERp44 is required for endocardial cushion development by regulating VEGFA secretion in myocardium
Source: Cell Prolif. 2022 Jan 28;55(3):e13179. doi: 10.1111/cpr.13179 (PMC8891561; doi:10.1111/cpr.13179)
Supplement: Supplementary file 9 — Table S3 [file CPR-55-e13179-s002.docx]

**Table S3. Sequence information of sgRNA for ERp44 knockout.**

| **Primers** | **Sequence** |
| --- | --- |
| ERp44-sgRNA-F | TTGATGTAAGTTACTTATGG |
| ERp44-sgRNA-R | CCATAAGTAACTTACATCAA |
